# Degradation of plant peroxisomes by autophagy

**DOI:** 10.3389/fpls.2014.00139

**Published:** 2014-04-08

**Authors:** Han Nim Lee, Jimi Kim, Taijoon Chung

**Affiliations:** Department of Biological Sciences, Pusan National UniversityBusan, South Korea

**Keywords:** autophagy-related, Atg, peroxisome-associated protein degradation, hydrogen peroxide, glyoxylate cycle enzymes

## Abstract

Peroxisomes play a critical role in many metabolic pathways during the plant life cycle. It has been proposed that the transition between different types of peroxisomes involves the degradation of obsolete peroxisomal enzymes via proteolytic activities in the peroxisome matrix, the cytosol, or the vacuole. Forward and reverse genetic studies recently provided evidence for autophagic degradation of peroxisomes in the vacuole of *Arabidopsis* seedlings. Here, we briefly review a model of pexophagy, or selective autophagy of peroxisomes, in plant cells.

## PLANT PEROXISOMES: TYPES, TRANSITION, AND PROTEIN DEGRADATION

Plant peroxisomes are versatile organelles that participate in many metabolic pathways such as fatty acid β-oxidation and photorespiration (reviewed by Hu et al., 2012). In addition to the enzymes needed for these pathways, peroxisomes contain antioxidant enzymes, for example, catalase, to protect plants from oxidative damage, since hydrogen peroxide is generated from fatty acid β-oxidation and photorespiration and other oxidation reactions in the peroxisome.

Peroxisomes are dynamic organelles with the capacity to change their appearance, their association with other organelles, and their enzyme composition. These changes depend on the developmental program and metabolic needs of the cell. For example, when oil-storing seeds such as cucumber (*Cucumis sativus*) and *Arabidopsis* (*Arabidopsis thaliana*) germinate, peroxisomes contain the glyoxylate cycle enzymes. The enzymes are needed for the consumption of acetyl-CoA (the product of fatty acid β-oxidation) for synthesis of organic acids that can be used to generate sugars by gluconeogenesis (reviewed by Pracharoenwattana and Smith, 2008). These seedling peroxisomes, formerly called glyoxysomes, are closely associated with lipid bodies supplying fatty acids (Trelease et al., 1971). When the seedlings are exposed to light, peroxisomal glyoxylate cycle enzymes, such as isocitrate lyase (ICL) and malate synthase (MLS), are rapidly degraded and enzymes involved in photorespiration accumulate. These peroxisomes are referred to as leaf peroxisomes. The change in peroxisomal enzyme composition may result from the transition of seedling peroxisomes to leaf peroxisomes (the “one-population model”), rather than from the degradation of seedling peroxisomes and the formation of new leaf peroxisomes (the “two-population model”; Beevers, 1979; Nishimura et al., 1996). Light also triggers changes in the position of peroxisomes. Seedling peroxisomes are associated with lipid bodies, while leaf peroxisomes are positioned near chloroplasts (Trelease et al., 1971; Gruber et al., 1973) from which glycolate, a photorespiration intermediate, enters the peroxisome for oxidation. Interestingly, a reverse transition from leaf peroxisomes to peroxisomes containing ICL may occur during starvation and organ senescence (reviewed by Nishimura et al., 1996; Pracharoenwattana and Smith, 2008).

**FIGURE 1:**
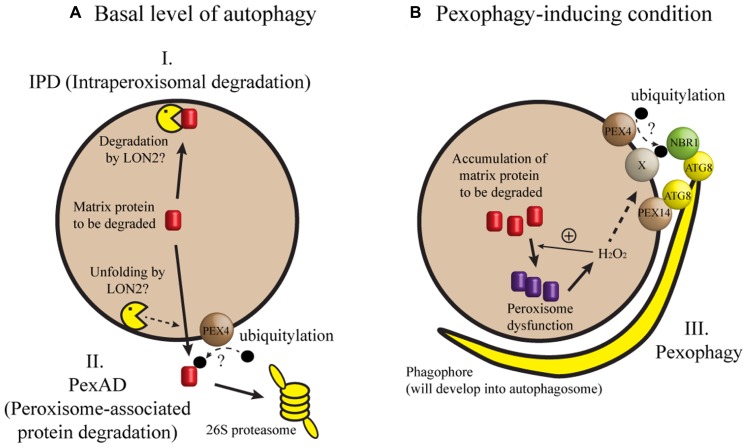
**A model of peroxisomal protein degradation in plant cells. (A)** When autophagy occurs at a basal level or when a core *ATG* gene is missing, degradation of matrix proteins like ICL and MLS may depend on intraperoxisomal degradation (IPD) or peroxisome-associated protein degradation (PexAD). **(B)** Pexophagy may be induced by loss of *LON2*, inactivation of IPD and PexAD, or a developmental process in which peroxisomes are past a critical level of oxidative stress (for example, intensive fatty acid β-oxidation during early seedling growth). Under pexophagy-inducing conditions, phagophore initiates, expands, and forms the autophagosome to sequester and target the peroxisome to the vacuole for degradation. In this model, hydrogen peroxide (H_2_O_2_) is shown as a sole matrix-derived induction signal for pexophagy, although we cannot exclude the possibility of additional induction signals. Refer to the main text for possible contributions of PEX4, PEX14, NBR1, and ATG8 proteins to target recognition. Broken arrows indicate highly speculative steps.

When seedling peroxisomes are transformed to leaf peroxisomes, obsolete ICL and MLS must be degraded. In recent studies, three mechanisms have been proposed for the degradation of these proteins during post-germinative growth of *Arabidopsis* seedlings (**Figure 1**). In one mechanism [herein designated intraperoxisomal degradation (IPD)], it is proposed that peroxisomal proteins are degraded by resident proteases. However, known peroxisomal proteases, which include Lon-related protease 2 (LON2), have not been implicated in full degradation of peroxisomal matrix proteins (Lingard and Bartel, 2009). This argument was mainly based on the observation that ICL and MLS levels in *lon2* mutant were not higher than those in wild-type (Lingard and Bartel, 2009; Burkhart et al., 2013). According to a second mechanism, obsolete proteins are retranslocated from peroxisomes and degraded in the cytosol by the 26S proteasome. During this process, called peroxisome-associated protein degradation (PexAD), the proteins are polyubiquitylated before they are recognized by the proteasome, analogous to ER-associated protein degradation (ERAD; reviewed by Smith et al., 2012). The possibility of polyubiquitylation is supported by a survey of *Arabidopsis* ubiquitylome, in which ICL was identified as a ubiquitylated protein (Kim et al., 2013a). Furthermore, the *PEROXIN4* (*PEX4*) gene, which may be involved in ubiquitylation, is necessary for the degradation of ICL and MLS (Zolman et al., 2005; Lingard et al., 2009). A third mechanism for peroxisomal degradation is pexophagy, a selective type of autophagy in which peroxisomes are targeted to the vacuole.

Pexophagy and its mechanism are well described in methylotrophic yeast and to a lesser extent in mammalian cells (reviewed by Till et al., 2012). Pexophagy typically removes obsolete or damaged peroxisomes. For example, peroxisomes are proliferated when methylotrophic yeast is grown in methanol, and excess peroxisomes are eliminated by pexophagy when methanol is replaced by other carbon source. Two types of pexophagy are known in yeast: in macropexophagy, peroxisomes are sequestered by the phagophore (**Figure 1**) and subsequently targeted to the vacuole, and micropexophagy occurs when peroxisomes are directly engulfed by the vacuolar membrane (Till et al., 2012). It was found that many core *Autophagy-related* (*Atg*) genes are required for pexophagy in yeast and mouse. Despite significant progress in our understanding of plant autophagy (reviewed by Floyd et al., 2012; Li and Vierstra, 2012), direct evidence for pexophagy in plant cells has not been available until recently and will be discussed herein.

## DEGRADATION OF PEROXISOMES BY AUTOPHAGY IN *ARABIDOPSIS*

Early electron microscopy studies rarely include snapshots of autophagic degradation of peroxisomes in plant cells. However, there is a published example of autophagic vacuoles located near peroxisomes in castor bean endosperm, taken approximately 6 days after germination (Vigil, 1970). These snapshots alone were not sufficient for definitive evidence of plant pexophagy and required confirmation by immunoelectron microscopy and three-dimensional electron tomography.

A recent study employing a genetic suppressor screen provides evidence for pexophagy in plants. This was done by Dr. Bonnie Bartel’s group at Rice University in an attempt to identify the molecular function and targets of the LON2 protease (Farmer et al., 2013). The investigators screened for mutations suppressing *lon2* phenotypes of defective β-oxidation activity and incomplete processing of peroxisome targeting signal (PTS) 2. In addition to the phenotypes, *lon2* mutant cells had abnormally large spherical structures labeled by green fluorescent protein tagged with PTS (GFP-PTS), a widely used peroxisomal matrix marker. In contrast, the wild-type cells had small GFP-PTS puncta. Cloning of mutant genes that code for the *lon2* suppressors resulted in the identification of several alleles of *atg* genes, specifically *atg2*, *atg3*, and *atg7*, two of which had been previously shown to cause defective autophagy (Doelling et al., 2002; Inoue et al., 2006). Double mutants of *lon2 atg2*, *lon2 atg3*, and *lon2 atg7* all had normal β-oxidation and PTS2 processing, and had small GFP–PTS puncta. Moreover, endogenous ICL and MLS were stabilized in the double mutants, but not significantly in *atg* single-mutant seedlings. Farmer et al. (2013) presented a model in which autophagy removes a fraction of peroxisomes in wild-type *Arabidopsis* seedlings, while peroxisomal defects in *lon2* mutation induce pexophagy (**Figure 1B**). Similar results were obtained by Dr. Mikio Nishimura’s group at National Institute for Basic Biology, Japan (Goto-Yamada et al., 2014). Lack of autophagy in the *lon2* background appears to prevent the double-mutant seedlings from losing small peroxisomes, leading to the suppression of *lon2* phenotypes (Bartel et al., 2014; Goto-Yamada et al., 2014). Although the precise function of LON2 in ICL and MLS degradation has yet to be defined, data described in Farmer et al. (2013), Goto-Yamada et al. (2014) indicate that LON2 protease plays a pivotal role in the IPD, unfolding, and/or translocation of misfolded peroxisomal proteins (**Figure 1**).

A role for pexophagy in ICL and MLS degradation was also demonstrated in a reverse genetics study performed in our laboratory (Kim et al., 2013b). 5-day-old wild-type hypocotyls had approximately 50% fewer peroxisomes than 3-day-old hypocotyls, while the reduction was about 20% in *atg7* hypocotyls. Degradation of ICL and MLS was delayed in *atg7* hypocotyls, but this stabilization effect was not obvious at the whole-seedling scale. Consistent with the observation that phenotypes were more obvious in hypocotyls than in the whole seedling, *ATG7* transcription appeared to be induced preferentially in hypocotyls (Kim et al., 2013b). Thus, autophagy during seedling growth may be spatiotemporally controlled to promote degradation of peroxisomes. A relatively low level of autophagic activity in cotyledons and roots would explain why Farmer et al. (2013), Goto-Yamada et al. (2014) failed to detect stabilization of ICL and MLS in *atg7* and *atg2* single mutant seedlings. Finally, Kim et al. (2013b) reported *ATG7-* dependent degradation of peroxisomes in the central vacuole and observed autophagic puncta overlapping with peroxisomal markers.

As seedlings mature, pexophagy may have a role in peroxisomal quality control. This suggestion is supported by the results from another forward genetic screen performed by Dr. Mikio Nishimura’s group (Shibata et al., 2013). These authors identified mutants with aggregated peroxisomes and showed that the *atg2, atg7*, and *atg18a* mutations were responsible for aggregation. In line with the findings of Farmer et al. (2013), the *atg2* mutation did not affect peroxisome function. However, leaves from 3-week-old *atg2* mutant plants accumulated more peroxisomal proteins than the wild-type control, but had the same amount of mitochondrial and chloroplast proteins as leaves from wild-type plants, suggesting selective degradation of peroxisomal proteins by autophagy. Shibata et al. (2013) found that exogenously supplied hydrogen peroxide induced peroxisome aggregation. The aggregated peroxisomes in *atg2* mutants were highly oxidized and contained a high level of inactive catalase. More recently, Yoshimoto et al. (2014) observed a phagophore-like structure that formed near the aggregated peroxisomes in *atg2* leaves. These observations suggest that hydrogen peroxide is an induction signal for pexophagy, a process that aids in the disposal of damaged peroxisomes in the cell (**Figure 1B**).

## FUTURE RESEARCH PERSPECTIVES

We can identify several questions concerning pexophagy in plant cells. First, why have there been few ultrastructure images suggestive of pexophagy in plant cells? The scarcity may be due to rapid targeting of autophagic vesicles to the vacuole (Zhuang et al., 2013). This possibility is supported by the observation of the phagophore-like structures in *atg2*, where autophagosome formation may not be completed (Yoshimoto et al., 2014). In addition, the scarcity may result from a small developmental window in which seedling peroxisomes are rapidly transformed to leaf peroxisomes, as our study suggested (Kim et al., 2013b).

Another important question concerns the selectivity of autophagy. While autophagy in mature leaves may be selective for peroxisomes over mitochondria or plastids (Shibata et al., 2013), such selectivity was not clearly demonstrated in hypocotyls. A more quantitative tool to assess selectivity of autophagy in plant cells will be useful, and would apply to other types of selective autophagy, too.

Three of the studies mentioned here (Farmer et al., 2013; Kim et al., 2013b; Goto-Yamada et al., 2014) focused on peroxisome transition in young seedlings, while mature plants were used for the analysis of leaf peroxisomes in two other papers (Shibata et al., 2013; Yoshimoto et al., 2014). Nevertheless, these studies all underscore a role for autophagy in homeostasis of peroxisome number. Is there any unifying concept from the studies? It seems that the transition of seedling peroxisomes to leaf peroxisomes involves aggregation of small, highly oxidized peroxisomes that contain damaged or misfolded enzymes. In fact, aggregated peroxisomes were accumulated in the mesophyll cells of young *atg2* seedlings (Goto-Yamada et al., 2014) and possibly in *atg7* hypocotyl cells (Kim et al., 2013b). Hydrogen peroxide has been proposed as a signal for autophagy in plants (reviewed by Pérez-Pérez et al., 2012; Hackenberg et al., 2013). In support of this proposal, *cat2* seedlings lacking a detectable level of catalase showed accelerated degradation of ICL and MLS (Lingard et al., 2009; see **Figure 1B**) compared to wild-type seedlings. Future work should clarify whether hydrogen peroxide acts as an upstream signal for both general and selective autophagy.

What proteins are necessary for recognizing peroxisomes targeted for autophagy? In methylotrophic yeast, a pexophagy receptor Atg30 bridges the molecular interaction between an autophagic complex and the peroxisomal proteins Pex3 and Pex14 (Farré et al., 2008; Zutphen et al., 2008). In mammalian cells, Pex14 interacts with LC3, an Atg8 homolog (Hara-Kuge and Fujiki, 2008). In addition, p62 and Neighbor of BRCA1 gene 1 (NBR1) may form a bridge between an ubiquitylated peroxisomal protein and LC3 (Kim et al., 2008; Deosaran et al., 2013). Intriguingly, an *Arabidopsis* ortholog of yeast *Pex14* was identified from a genetic screen for mutants that showed stabilization of peroxisomal markers (Burkhart et al., 2013). It remains to be seen whether molecular interaction leading to pexophagy is conserved among distant eukaryotes (**Figure 1**).

## Conflict of Interest Statement

The authors declare that the research was conducted in the absence of any commercial or financial relationships that could be construed as a potential conflict of interest.
